# Reframing precision nutrition in irritable bowel syndrome: a mechanism-informed conceptual framework for responder prediction and clinical translation

**DOI:** 10.3389/fimmu.2026.1809221

**Published:** 2026-05-29

**Authors:** Ya Zhou, Zhen Li, Yuzhou Chu, Zhijia Zhou, Tao Zhang, Ning Yi, Wuquan Sun, Juntao Yan, Zhen Yan, Anning Zhu

**Affiliations:** 1 Department of Tuina, Yueyang Hospital of Integrated Traditional Chinese and Western Medicine, Shanghai University of Traditional Chinese Medicine, Shanghai, China; 2 Department of Endocrinology, The Second Affiliated Hospital, Zhejiang University School of Medicine, Hangzhou, China

**Keywords:** biomarkers, clinical validation, gut microbiome, irritable bowel syndrome, low-FODMAP diet, machine learning, metabolomics, multi-omics

## Abstract

**Background:**

The low-Fermentable Oligosaccharides, Disaccharides, Monosaccharides and Polyols (FODMAP) diet is widely used for irritable bowel syndrome (IBS), but response varies markedly across patients. This heterogeneity has shifted the field from testing average efficacy toward forecasting individual benefit and translating microbiome science into practical precision-nutrition tools.

**Methods:**

We present a conceptual analysis grounded in evidence mapping from human IBS studies that paired dietary interventions (primarily low-FODMAP pathways) with baseline microbiome and/or multi-omics measurements. Findings are organized within a “microbiome-to-model” roadmap that specifies responder endpoints, candidate data layers (taxa, functions, metabolites and volatile signatures), modeling choices, and the validation and implementation requirements needed for clinical decision support.

**Results:**

Three recurring signals emerge across cohorts. Baseline microbial ecology can stratify response, but taxonomic features alone often fail to transport across studies. Functional readouts, including metabolites and volatile signatures, are closer to symptom mechanisms and can improve interpretability; however, clinical deployment is still limited by endpoint heterogeneity, imperfect exposure and adherence measurement, batch effects, and insufficient external validation and calibration.

**Conclusion:**

IBS is well suited for microbiome-informed responder prediction, provided that models are developed with deployment in mind. Progress will depend on validation-first study designs, harmonized responder endpoints and adherence capture, robust multi-omics pipelines, and biologically interpretable decision rules that can be prospectively tested and monitored for temporal instability in real-world care.

## 1 Introduction: why microbiome-informed precision nutrition is becoming unavoidable in IBS

### 1.1 IBS as a disorder of gut–brain interaction with high heterogeneity and unmet needs

Irritable bowel syndrome (IBS) is a prototypical disorder of gut–brain interaction (DGBI)—meaning it exemplifies the category of functional gastrointestinal conditions in which symptoms arise from dysregulated gut–brain signaling rather than structural or biochemical pathology detectable by routine investigation—in the Rome IV framework. Although it is defined clinically by recurrent abdominal pain linked to defecation and accompanied by altered stool frequency and/or form, its biological correlates extend well beyond motility. Across mechanistic and human studies, IBS has been associated with visceral hypersensitivity, mucosal immune activation, epithelial barrier disruption, altered gut microbial ecology, and differences in central processing of visceral inputs—features that collectively point to multi-level dysregulation rather than a single dominant lesion (1–3). This matters because the burden is not marginal: Rome Foundation epidemiology suggests that more than 40% of individuals meet criteria for at least one DGBI, with tangible impacts on quality of life and healthcare utilization (4).

Within IBS, heterogeneity is not a secondary nuance; it is the central clinical problem. Patients differ by bowel-habit pattern—IBS with predominant diarrhea (IBS-D), IBS with predominant constipation (IBS-C), and IBS with mixed bowel habits (IBS-M)—but also by dietary triggers, stress exposure, medication histories (notably antibiotics), and baseline microbial configuration. As a result, generic dietary recommendations predictably yield mixed outcomes: they may relieve symptoms for some while offering little for others, even when diagnostic labels appear similar (5, 6).

Rome IV implicitly reframes this dilemma. If IBS represents an emergent phenotype generated by host–microbe–diet interactions along the gut–brain axis, then patient-to-patient variation is expected rather than anomalous (1, 2). The practical consequence is straightforward: population-average dietary effects will remain clinically blunt unless responders can be identified prospectively.

### 1.2 Diet as a primary modulator of IBS symptoms—and a major source of response variability

Dietary intervention is often the first management lever in IBS, partly because symptoms are commonly meal-related, and partly because diet is a dominant upstream driver of microbial substrate supply and metabolite output. Among dietary strategies, the low-Fermentable Oligosaccharides, Disaccharides, Monosaccharides and Polyols (FODMAP) approach has some of the most consistent trial evidence for symptom improvement, yet its effects are not uniform across patients. Controlled trials demonstrate symptom reductions relative to habitual diets, but benefits can overlap with those achieved by structured traditional IBS dietary advice, implying that more than one dietary pathway can be effective—and that the “best” option may depend on the individual (7, 8).

The mechanistic logic of diet response is plausible: reducing fermentable substrate can alter luminal distension signals; changing macronutrient patterns may influence bile-acid handling; and broader dietary shifts can remodel microbial metabolic pathways. However, symptom improvement is not synonymous with ecological benefit. Fermentable carbohydrate restriction has been linked to reduced luminal *Bifidobacteria* even as symptoms improve, illustrating that clinical gain may coexist with a narrower microbial ecology (9). This tension sharpens the rationale for precision approaches. If only a subset benefits substantially, and if some baseline microbial configurations are more prone to undesirable ecological shifts, then “who should do what diet” becomes an essential translational question—especially when long-term care requires reintroduction and maintenance rather than indefinite restriction.

### 1.3 From “diet works” to “for whom and why”: microbiome subtypes, multi-omics signals, and AI-assisted personalized diets

The field is increasingly moving from demonstration to discrimination: not whether diet can help IBS, but which patients are likely to benefit from which dietary strategy, and why. A key inflection point has been evidence that baseline microbiota patterns can stratify dietary response. In a landmark study, two gut microbiota subtypes were identified among IBS patients with distinct responses to low-FODMAP intervention, supporting the view that microbial community state can modify treatment effects rather than simply follow dietary change (10). Once effect modification is acknowledged, multi-omics integration becomes less optional and more methodological: taxonomic composition alone is unlikely to capture the relevant variance, whereas functional readouts such as metabolites, alongside clinical phenotypes and dietary exposure features, are better aligned with mechanism and thus with prediction.

In parallel, microbiome-informed personalization has begun to move from concept to clinical testing. A pilot study reported that an AI-driven personalized diet aimed at modulating the microbiome was associated with reduced IBS symptom severity—particularly in IBS-M—together with shifts in selected taxa (11). More decisively for translation, a multicenter randomized controlled trial compared a microbiome-based AI-assisted personalized diet directly with a low-FODMAP strategy, establishing feasibility for head-to-head evaluation against guideline-adjacent care and providing higher-level evidence that algorithm-guided personalization can be assessed as an intervention rather than a retrospective model (12).

These developments motivate the present conceptual analysis. IBS is diet-sensitive and heterogeneous, and the literature is now rich enough that the limiting factor is no longer “evidence scarcity,” but misalignment: between mechanisms and measurable proxies, between prediction targets and clinical decisions, and between internal performance and cross-cohort transportability. The central task, therefore, is to translate mechanistic understanding into responder-prediction pipelines that remain clinically usable, robust to confounding (including medication exposures and dietary reporting noise), and stable across populations and settings.

Schematic overview of a mechanism-to-measurement-to-modeling pathway designed to explain and reduce the variable efficacy of dietary therapies in irritable bowel syndrome (IBS). Key symptom-relevant mechanism modules include fermentation and gas-related distension, bile acid dysregulation, and barrier vulnerability with neuro-immune amplification. These mechanisms are connected to measurable biomarkers—microbial community features and functional readouts such as metabolites and volatile organic compounds (VOCs)—to support mechanistically grounded stratification and responder prediction. The lower panel emphasizes that taxonomic signatures may not generalize well across cohorts, whereas functional readouts are closer to symptom biology and can improve interpretability The right panel summarizes implementation bottlenecks (inconsistent responder definitions, inadequate exposure/adherence measurement, and batch effects/leakage risk) and a translational roadmap centered on a minimum viable dataset, an expanded R&D dataset, and 4–6 week reevaluation with protocol switching using standardized endpoints. The right panel summarizes implementation bottlenecks (inconsistent responder definitions, inadequate exposure/adherence measurement, and batch effects/leakage risk) and a translational roadmap centered on a minimum viable dataset, an expanded R&D dataset, and 4–6 week reevaluation with protocol switching using standardized endpoints.

## 2 Scope and methods

### 2.1 Scope and objectives of this conceptual analysis

This conceptual analysis addresses microbiome-informed precision nutrition in IBS with a narrow translational emphasis: how to anticipate individual response to dietary intervention, rather than restating population-average efficacy. We center on low-FODMAP–based care because it is widely implemented and supported by relatively mature mechanistic and multi-omics evidence. Where relevant to model design and clinical deployment, we also consider emerging microbiome-assisted personalized diet strategies.

Our objectives are threefold. First, we organize evidence that links diet-related symptom changes to baseline microbial ecology, microbial functional outputs, and host susceptibility factors that plausibly modify treatment effects. Second, we examine how these layers have been operationalized in statistical and machine-learning approaches for responder prediction. Third, we identify recurring barriers that undermine transportability and clinical usability, including endpoint heterogeneity, dietary exposure and adherence mismeasurement, batch effects across assays and pipelines, and the scarcity of targeted external validation.

### 2.2 Literature search and selection

We conducted an evidence-mapping exercise guided by PRISMA 2020 and PRISMA-ScR reporting principles to make the evidence base and methodological gaps transparent, without undertaking a formal quantitative meta-analysis (13, 14). Searches were performed primarily in PubMed/MEDLINE and complemented by citation tracking from consensus statements, pivotal trials, and mechanistic studies most relevant to responder prediction.

Search strings combined controlled vocabulary and free-text terms spanning IBS (“irritable bowel syndrome,” “functional bowel disorders”), dietary interventions (“low FODMAP,” “dietary restriction,” “personalized diet”), microbiome and functional profiling (“gut microbiota,” “metagenomics,” “metabolomics,” “volatile organic compounds”), host mechanisms (“intestinal permeability,” “mast cells,” “mucosal immunity”), and computational approaches (“machine learning,” “artificial intelligence,” “prediction model”). We prioritized human evidence, including randomized controlled trials, pragmatic trials, and well-characterized observational cohorts. Non-IBS studies were included only when they offered methodological guidance with clear transferability to IBS research (for example, standardization of breath testing or harmonization of outcome definitions). Searches were conducted from database inception through March 2025; citation tracking extended to studies identified through February 2026. Inclusion criteria required human IBS studies pairing dietary intervention or microbiome characterization with prospectively collected, validated symptom outcomes. Non-IBS studies were included only when they offered methodological guidance with direct transferability to IBS prediction research. Case reports, conference abstracts lacking peer-reviewed data, and non-English-language publications were excluded. Because this is a conceptual scoping analysis rather than a formal systematic review, a full PRISMA flow diagram is not generated; however, the key studies informing each evidence domain are catalogued in **Supplementary Table 1**.

To maintain a decision-oriented structure, evidence was organized by its relevance to feature design and care-pathway integration. Studies were grouped into dietary intervention strategies, microbiome composition and function, metabolomics and volatilomics, host susceptibility phenotypes, and predictive modeling. This organization provides a stable conceptual scaffold while allowing individual modules to be updated as the field evolves.

### 2.3 Operational definitions: diagnosis, outcomes, and responder status

IBS diagnosis and subtyping. IBS was defined as a disorder of gut–brain interaction diagnosed using symptom-based criteria with limited testing to exclude organic disease, consistent with the Rome IV framework (1, 2). Where earlier Rome iterations were used, diagnostic criteria and bowel-habit subtypes were extracted to support cross-study comparison.

Clinical endpoints. Heterogeneity in outcome definitions represents a major barrier to both mechanistic inference and model validation. We therefore prespecified a hierarchy of clinically meaningful endpoints commonly used in IBS trials. Symptom severity was primarily framed using the IBS Severity Scoring System (IBS-SSS), a validated instrument widely applied for monitoring disease trajectory and treatment response (15). Binary “relief-based” endpoints were also considered, recognizing their historical use but also their sensitivity to baseline severity and contextual factors (16, 17).

Regulatory alignment. For studies aiming at clinically actionable prediction, we paid particular attention to regulatory guidance. The U.S. Food and Drug Administration emphasize improvement in abdominal pain and bowel habit as core domains for IBS treatment evaluation, while the European Medicines Agency provides complementary recommendations on endpoint selection and population definition (18, 19). Where possible, study-specific outcomes were mapped to these domains to facilitate interpretation and future model harmonization.

### 2.4 Standardized phenotyping relevant to diet–microbe mechanisms

Diet-related symptoms in IBS can reflect carbohydrate malabsorption, altered fermentation dynamics, and disordered transit. Breath testing is therefore frequently used to phenotype lactose or fructose malabsorption and to explore methane-associated phenotypes. However, methodological variability in breath testing can substantially affect classification and downstream interpretation.

To mitigate this source of heterogeneity, we interpreted breath-test–based evidence considering established consensus recommendations. The Rome Consensus Conference provides foundational guidance on indications and methodology for hydrogen breath testing in gastrointestinal disorders (20). The North American Consensus further standardizes hydrogen- and methane-based breath testing, with specific recommendations on substrates, dosing, preparation, and interpretation (21). More recently, a European guideline has integrated evidence and expert consensus across adult and pediatric populations, offering a comprehensive framework for harmonized breath testing in both clinical practice and research (22).

In the context of precision nutrition, these guidelines are not peripheral. An important limitation that applies throughout this framework is that hydrogen and methane breath test results do not always correlate with symptom onset or severity at the individual level. The relationship between measurable gas production and symptomatic response is modified by transit time, host sensitivity thresholds, and colonic flora composition, among other factors. Breath test findings should therefore be interpreted as one mechanistic input within a multi-feature model, not as a standalone criterion for dietary selection. Misclassification of malabsorption states or methane-associated phenotypes introduces systematic measurement error into dietary response labels, undermining both mechanistic inference and predictive model performance.

### 2.5 Rationale for a precision nutrition framing: efficacy, sustainability, and personalization constraints

While low-FODMAP restriction has strong short-term evidence, longer-term management requires reintroduction and personalization to avoid unnecessary restriction and potential adverse impacts on nutrient intake and microbiome ecology. Contemporary clinical reviews emphasize that structured implementation (often dietitian-led), staged reintroduction, and individualized trigger identification are essential for sustainable care pathways (23). This practical reality reinforces the need for precision strategies: responder prediction should not merely identify “who improves,” but also “who can improve with minimal restriction,” and “who requires adjunctive strategies” (e.g., targeted fibers, probiotics, or algorithm-guided personalization) to balance symptom control with ecological and nutritional adequacy.

## 3 Results

### 3.1 Dietary interventions for IBS: efficacy tiers, implementation constraints, and responder heterogeneity

#### 3.1.1 Low-FODMAP dietary strategy: what works, for whom, and at what cost

Randomized controlled trials and controlled feeding studies consistently support the low-FODMAP strategy as an effective approach for reducing global symptom burden in IBS, particularly abdominal pain and bloating (7, 8, 24). However, even under structured delivery, a substantial proportion of patients do not achieve clinically meaningful improvement (defined here as a ≥50-point reduction in IBS-SSS, consistent with established minimally important difference thresholds (15)), and treatment effects can overlap with those observed under structured “traditional IBS dietary advice” in some trials (8); however, Eswaran et al. (2016) reported a significant between-group difference in adequate symptom relief (52% vs 21%, p=0.0001) and abdominal pain response in favor of the low-FODMAP diet compared with modified NICE guidance, underscoring that the magnitude of benefit may differ meaningfully depending on the comparison arm and outcome definition (24). These findings indicate that low-FODMAP efficacy is conditional rather than universal.

From a mechanistic perspective, low-FODMAP restriction reduces the intestinal delivery of poorly absorbed carbohydrates, thereby attenuating osmotic water shifts and colonic fermentation that contribute to luminal distension and symptom perception (25, 26). Importantly, symptom improvement does not necessarily imply restoration of microbial ecology. Fermentable carbohydrate restriction has been associated with reductions in beneficial taxa such as bifidobacteria, raising concerns regarding prolonged or indiscriminate restriction (9). This trade-off between short-term symptom relief and potential ecological cost underscores why responder identification is clinically relevant: patients unlikely to benefit may incur unnecessary dietary restriction without commensurate symptom gain.

#### 3.1.2 Low-FODMAP as a process rather than a static diet: restriction, reintroduction, and personalization

Current best practice conceptualizes the low-FODMAP approach as a multistep process rather than a single dietary state. This process typically involves an initial short-term restriction phase, followed by structured reintroduction of specific FODMAP groups to identify individual triggers, and finally long-term personalization to maintain symptom control while preserving dietary diversity and nutritional adequacy (23).

This distinction has important implications for both clinical trials and responder-prediction research. Studies that define “low-FODMAP response” without adequate assessment of adherence, reintroduction, or long-term dietary quality may conflate biological response with implementation effects, such as incomplete restriction, reporting bias, or early discontinuation. From a precision-nutrition perspective, the clinically relevant questions are therefore not limited to whether a patient responds during short-term restriction, but also whether symptom control can be sustained with minimal ongoing restriction and whether alternative strategies may be preferable for non-responders.

Dietitian-led delivery and structured education remain central to minimizing variability in implementation and improving reproducibility across studies and clinical settings (23).

#### 3.1.3 Beyond low-FODMAP: alternative dietary paradigms and implications for heterogeneity

One reason IBS represents a fertile setting for precision nutrition is that multiple dietary paradigms demonstrate efficacy, likely through partially distinct biological mechanisms. Comparative trials suggest that symptom improvement can be achieved via different dietary routes, reinforcing the concept that IBS dietary management resembles a selection problem under heterogeneity rather than a single optimal solution.

Structured traditional IBS dietary advice, including guidance on meal regularity, fiber modification, and reduction of trigger foods, has shown symptom improvements comparable to low-FODMAP restriction in some trials, emphasizing that not all responders require extensive carbohydrate exclusion (8, 24). More recently, less restrictive dietary patterns have been explored. A randomized trial comparing a Mediterranean-style diet with a low-FODMAP diet in non-constipated IBS demonstrated symptom improvement in both groups, with greater reduction in IBS-SSS under low-FODMAP restriction, but also highlighted differences in feasibility and long-term acceptability (27).

Other comparative studies further expand the intervention landscape. A pragmatic randomized trial comparing low-FODMAP advice, a low-carbohydrate diet, and optimized medical therapy supports the view that multiple first-line strategies are reasonable, with differential benefit across patients (28). Gluten-related strategies provide another example of heterogeneity: controlled trials suggest that a subset of patients with IBS, particularly IBS-D, may benefit from gluten-free diets, with evidence that genetic or metabolic profiles modify response (29, 30). Similarly, a starch- and sucrose-reduced diet (SSRD) has demonstrated symptom improvement in randomized trials, accompanied by changes in inflammatory and metabolic mediators (31, 32).

Collectively, these findings argue against a universal dietary hierarchy in IBS. Instead, they support a precision framework in which baseline physiological and microbial features guide selection among multiple evidence-based dietary options. Importantly, the prediction architecture proposed in this framework is not low-FODMAP-specific. The data layer structure—from phenotype characterization through mechanistic proxies to model-generated treatment probabilities—is designed to be diet-agnostic at the encoding level. Low-FODMAP currently serves as the primary anchor because it has the most developed mechanistic and multi-omics evidence base; however, as evidence matures for SSRD, Mediterranean-style, gluten-free, and microbiome-informed AI-personalized strategies, the same framework can incorporate these as competing treatment arms. The limiting factor is not the architecture, but the availability of adequately characterized baseline-response datasets for each strategy.

#### 3.1.4 Guideline perspectives and real-world delivery constraints

Clinical guidelines increasingly recognize diet as a core component of IBS management while emphasizing the importance of appropriate delivery and follow-up. The American College of Gastroenterology recommends a limited trial of a low-FODMAP diet for global IBS symptoms, with the caveat that implementation should ideally involve trained dietary professionals to support reintroduction and personalization and to mitigate risks associated with prolonged restriction (33).

From a translational standpoint, guideline-era practice exposes a key challenge for precision nutrition research: many patients initiate dietary restriction in an unstructured or self-directed manner (34), resulting in substantial exposure misclassification. This variability complicates interpretation of both clinical outcomes and predictive models. Consequently, studies aiming to develop or validate responder-prediction tools must prioritize standardized outcome measures, structured dietary protocols, and objective or semi-objective phenotyping wherever feasible (20–22, 33).

### 3.2 Mechanistic pathways linking diet, microbiome, and symptom generation in IBS

#### 3.2.1 FODMAP biology: osmotic water shifts and fermentation-driven luminal distension

The mechanistic basis for restricting fermentable carbohydrates in IBS lies in the handling of poorly absorbed short-chain carbohydrates within the gastrointestinal tract. These carbohydrates increase small-intestinal water content through osmotic effects and simultaneously enhance delivery of fermentable substrates to the colon, where microbial metabolism generates gas and distension signals that may be perceived as pain or bloating in susceptible individuals (35, 36). Direct physiological evidence supports this model: controlled feeding studies demonstrated that poorly absorbed short-chain carbohydrates increase the delivery of both water and fermentable substrates to the proximal colon, providing a concrete link between dietary composition and luminal conditions associated with symptom generation (35).

Experimental manipulation of dietary FODMAP content further showed that changes in fermentable carbohydrate exposure alter breath hydrogen and methane profiles and can reproducibly elicit gastrointestinal symptoms in IBS, consistent with fermentation dynamics acting close to the symptom-generating interface (36). These observations indicate that dietary effects in IBS are mediated less by nonspecific intolerance and more by quantifiable physical and metabolic processes within the intestinal lumen.

#### 3.2.2 Gas phenotype as a mechanistic axis: methane, transit slowing, and constipation-predominant features

Beyond total gas production, gas composition itself appears to influence gastrointestinal physiology. Methane (CH_4_), produced by enteric methanogens, has been shown experimentally to slow small-intestinal transit and enhance contractile activity of the small bowel, providing a mechanistic explanation for the association between methane-positive breath tests and constipation-predominant features in IBS (37).

This physiology has practical implications for dietary intervention. In patients with methane-associated transit slowing, reducing fermentable substrate load alone may not fully normalize bowel function if methane production and its downstream effects persist. From a modeling perspective, this distinction helps explain why symptom-based subtyping alone performs poorly as a predictor of dietary response and why mechanistic phenotypes derived from breath testing may improve both clinical interpretation and predictive accuracy.

#### 3.2.3 Microbial metabolites as symptom mediators and stratifiers: bile acids and serotonin signaling

##### 3.2.3.1 Bile acids define a clinically actionable IBS-D subgroup

Dysregulation of bile-acid (BA) metabolism has emerged as a distinct and treatable mechanism within diarrhea-predominant IBS. Studies demonstrate that a subgroup of IBS-D patients exhibits increased bile-acid synthesis or fecal bile-acid excretion, accompanied by physiological features that differ from those observed in IBS-D patients with normal bile-acid profiles (38). Independent genetic and physiological investigations further associate variation in bile-acid regulatory pathways with intestinal transit phenotypes, reinforcing the view that bile-acid biology represents a primary driver rather than a secondary consequence of diarrhea in these patients (39).

Clinically, bile-acid diarrhea has also been linked to increased intestinal permeability compared with IBS-D without bile-acid malabsorption, suggesting that excess bile acids may contribute to barrier stress and enhanced symptom signaling (40). Together, these findings support bile-acid dysregulation as a meaningful effect modifier within IBS-D that can influence both symptom expression and dietary responsiveness.

##### 3.2.3.2 Microbiota–mast cell–PGE_2_ regulation of SERT and 5-HT signaling

Serotonin (5-hydroxytryptamine, 5-HT) plays a central role in regulating intestinal motility and visceral sensitivity. A mechanistic study demonstrated that expression of the mucosal serotonin reuptake transporter (SERT) in IBS can be modulated by the gut microbiota via a mast cell–prostaglandin E_2_ (PGE_2_) signaling pathway, providing a direct mechanistic link between luminal microbial activity and host neurochemical regulation (41). This pathway offers a plausible explanation for how microbiome alterations may influence diarrhea and pain phenotypes without overt inflammation.

From the perspective of dietary intervention, these data highlight that microbial metabolites and host response pathways jointly determine symptom expression, and that modulation of the microbiome may preferentially affect motility and sensitivity in patients with specific neurochemical susceptibilities.

#### 3.2.4 Volatilomics as a functional bridge from fermentation ecology to clinical stratification

Volatile organic compounds (VOCs) and the fecal volatilome reflect the combined output of microbial metabolism and host substrate availability. Because they capture downstream metabolic activity rather than community composition alone, VOCs offer a functional readout that is closely linked to fermentation dynamics. Early work demonstrated that multivariate fecal VOC profiles can discriminate IBS-D from inflammatory bowel disease and healthy controls, highlighting their potential utility in a condition lacking objective diagnostic markers (42).

More recent studies extended this concept to dietary response. In IBS cohorts, microbiome-driven metabotypes identified through volatilomic profiling were associated with differential response to a low-FODMAP diet, with shifts in fecal VOC patterns tracking symptom improvement in responders (43). These observations position volatilomics as a promising intermediate phenotype linking diet, microbial metabolism, and clinical outcomes.

#### 3.2.5 Host barrier integrity and neuroimmune activation as amplifiers of luminal signals

Luminal stimuli alone are insufficient to explain symptom generation in IBS; host susceptibility plays a decisive role. Increased intestinal membrane permeability has been documented in a subset of patients with diarrhea-predominant IBS and is associated with greater symptom severity and heightened visceral pain sensitivity (44). Broader mechanistic analyzes indicate that multiple pathways contribute to barrier impairment in IBS, with permeability changes being particularly relevant in IBS-D (45).

More detailed epithelial studies reveal that barrier dysfunction is not uniform across IBS phenotypes. In mixed-type IBS, impaired tricellular tight-junction function and altered antigen uptake have been demonstrated using ex vivo approaches, underscoring the heterogeneity of epithelial alterations within the disorder (46). In parallel, neuroimmune interactions contribute to symptom amplification. Activated mast cells located near colonic nerves correlate with abdominal pain severity in IBS, implicating mast-cell mediators in the sensitization of enteric neural pathways (47).

Viewed together, these findings support a model in which luminal fermentation products and microbial metabolites act as triggers, while compromised barrier function and neuroimmune activation lower the threshold for symptom perception.

#### 3.2.6 Toward feature-ready mechanistic integration for predictive modeling

Across these mechanistic domains, a consistent pattern emerges: no single pathway adequately explains dietary response in IBS. Instead, response appears to reflect the interaction of microbial ecology, functional metabolic output, physical properties of luminal contents, and host susceptibility traits. Empirical evidence supports integrating microbiome subtypes associated with low-FODMAP response (48), functional outputs such as volatilomic signatures (43), physiological triggers related to osmotic load and fermentation (35, 36), and host factors including barrier integrity and neuroimmune activation (44–47).

This multi-layered organization also clarifies why predictive models often struggle with external validation. Each component is sensitive to contextual factors such as habitual diet, medication exposure, geography, and analytical pipelines. Emphasizing mechanism-proximal features and explicitly modeling host susceptibility as an effect modifier, rather than background measurement variability, may therefore improve both interpretability and transportability of responder-prediction frameworks.

### 3.3 Data layers for responder prediction: from microbial structure to functional and host-derived features

#### 3.3.1 Microbial composition profiles: utility and intrinsic limitations of taxonomic features

Early microbiome studies in IBS identified numerous taxonomic differences compared with healthy controls; however, reproducibility across cohorts has remained limited. This inconsistency reflects substantial heterogeneity in diagnostic criteria, background diet, geography, sequencing platforms, and analytical pipelines. Large systematic reviews and population-based studies have consequently cautioned against interpreting individual taxa as stable disease markers, as neither stool nor mucosal microbiota profiles consistently define IBS across unselected populations (49, 50).

A more informative approach has been to examine microbial composition in relation to *actionable outcomes* rather than case–control status. In this context, the identification of distinct IBS microbiota subtypes with differential response to a low-FODMAP diet demonstrated that baseline community structure can function as an effect modifier of dietary intervention (48). Related work linking microbial patterns to symptom severity, methane production, and reduced microbial richness further suggests that compositional data can capture broad ecological states relevant to physiology, even if fine-grained taxonomic signatures remain unstable (51).

Machine-learning methods applied to 16S rRNA gene sequencing or metagenomic data can extract multivariate patterns that are not apparent in univariate analyzes, but their apparent performance often depends heavily on cohort-specific characteristics and confounder structure (52, 53). From a predictive standpoint, microbial composition is therefore most useful when it delineates higher-level ecological configurations or subtypes that interact with diet, rather than when it is treated as a standalone predictor of response.

#### 3.3.2 Functional readouts and metabolomics: prioritizing what microbes do over who they are

Because taxonomic composition varies widely across populations, functional readouts that reflect microbial and host metabolism offer a more direct link to symptom-generating mechanisms. Metabolomic profiling has revealed alterations in multiple biochemical pathways in IBS, including bile-acid metabolism, short-chain fatty acid production, and amino acid–derived signaling molecules, while also highlighting the importance of analytical standardization and careful interpretation (54).

Evidence directly comparing functional and compositional predictors is particularly instructive. In a low-FODMAP intervention study, fecal and urinary metabolite profiles—but not gut microbiota composition—were able to predict symptom response, suggesting that biochemical outputs may capture diet responsiveness more faithfully than taxonomic snapshots (55). Controlled dietary challenge experiments further support this view, showing that FODMAP exposure alters bile-acid profiles and tryptophan- and phenolic-derived metabolites in ways that plausibly affect motility, epithelial signaling, and gut–brain communication (56).

From a modeling perspective, metabolite features occupy a dual role. At baseline, they characterize physiological states such as bile-acid–driven diarrhea or heightened fermentation load; after intervention, they can serve as intermediate readouts that improve interpretability and help distinguish true biological response from nonspecific symptom fluctuation. This property makes metabolomics particularly attractive for responder prediction in heterogeneous disorders such as IBS.

#### 3.3.3 Volatilomics as an intermediate phenotype linking fermentation ecology to symptoms

VOCs provide a functional window into microbial fermentation and substrate utilization. Fecal volatilome profiling integrates microbial activity with host substrate availability and therefore reflects processes that are temporally and mechanistically close to symptom generation. Early studies demonstrated that multivariate VOC profiles could distinguish IBS from inflammatory bowel disease and healthy controls, indicating sensitivity to disease-relevant metabolic states even in the absence of overt inflammation (42).

More recent work extended this concept to dietary response. In IBS cohorts, microbiome-driven metabotypes defined by volatilomic features were associated with differential response to a low-FODMAP diet, and responders exhibited distinct shifts in volatile profiles following intervention (43). These findings suggest that VOCs can function as intermediate phenotypes that bridge microbial ecology and clinical outcomes.

For prediction, volatilomics offers several practical advantages. Functional volatile signatures may generalize better across cohorts than taxonomic features that are sensitive to geography and sequencing methodology, and they are amenable to repeated, potentially noninvasive sampling. As such, VOC-based features are well suited to both baseline stratification and longitudinal monitoring of dietary response.

#### 3.3.4 Host susceptibility layers: barrier integrity, immune activation, and neuroimmune amplification

Dietary substrates and microbial metabolites do not inevitably produce symptoms; host susceptibility determines whether luminal signals cross the threshold for pain or bowel dysfunction. Among host-derived features, epithelial barrier integrity and neuroimmune activation are repeatedly implicated in IBS pathophysiology. Increased intestinal permeability has been documented in subsets of IBS, particularly IBS-D, and is associated with heightened visceral sensitivity and symptom severity (44, 45). More detailed epithelial studies reveal that barrier dysfunction is not uniform: in IBS-M, impaired tricellular tight-junction function and altered antigen uptake have been demonstrated, underscoring mechanistic heterogeneity across subtypes (46).

Neuroimmune interactions further shape symptom perception. Mast cell activation near enteric nerves correlates with abdominal pain severity, implicating mast cell mediators as amplifiers of neural signaling rather than mere epiphenomena (47). These host-derived traits provide a biological explanation for why similar luminal exposures result in divergent symptom experiences across patients.

In predictive models, host susceptibility features naturally function as moderators—variables that modify the direction or magnitude of treatment effects rather than lying on the causal pathway (57, 58). Explicitly modeling permeability and neuroimmune activation helps separate trigger intensity from individual sensitivity, improving interpretability and reducing unexplained variance that would otherwise undermine generalizability.

#### 3.3.5 Capturing dietary exposure and behavior: reducing label noise in responder prediction

Accurate responder prediction depends not only on biological features but also on reliable characterization of dietary exposure. In IBS studies, self-reported intake is vulnerable to recall bias and misclassification, particularly for fermentable carbohydrates, leading to outcome labels subject to substantial measurement error and misclassification that obscure biological associations. Standardized digital recall systems and structured dietary assessment tools provide a scalable means of improving intake measurement and aligning exposure with symptom trajectories (59).

Image-assisted food logging and automated dietary apps can further increase feasibility and temporal resolution, although their validity varies and must be evaluated against reference methods (60, 61). Beyond intake alone, digital health platforms designed for IBS management generate longitudinal data streams that include symptoms, stress, sleep, and adherence, offering opportunities to model dynamic diet–symptom relationships rather than static baseline associations (62, 63).

In practice, digital phenotyping is not ancillary to precision nutrition—it is often the component that determines whether a model learns meaningful biology or merely reflects systematic measurement artifact.

### 3.4 Artificial intelligence/machine learning modeling for IBS precision nutrition: from problem formulation to external validation and clinical deployment

#### 3.4.1 What exactly should an IBS “responder model” predict?

To date, most AI applications in IBS have focused on diagnosis, classification, or symptom-based subtyping rather than on treatment response. Recent systematic reviews conclude that while AI approaches show promise across screening, phenotyping, and management, their translational maturity varies widely depending on study design, endpoints, and data provenance (64). For precision nutrition, however, the clinically meaningful targets are narrower and decision-oriented.

In practice, responder models typically aim to predict one of three outcomes. The first is a binary response to a predefined dietary intervention—such as a structured low-FODMAP pathway, a starch- and sucrose-reduced diet (SSRD), or an AI-personalized dietary program—anchored to validated clinical endpoints, including established IBS-SSS thresholds or composite outcomes aligned with regulatory guidance (18, 19). A second, increasingly relevant task is treatment selection: predicting which dietary strategy among several evidence-based first-line options is most likely to benefit a given individual, reflecting real-world scenarios in which multiple diets demonstrate efficacy but with heterogeneous effects (28). A third approach focuses on mechanism-linked intermediate outcomes, such as shifts in breath gas phenotypes, bile-acid modules, or VOC/volatilome profiles, which can improve interpretability and robustness when symptom labels are noisy or context dependent (43, 56).

A key methodological implication is that “diet response” is not a single phenotype. It is intervention-specific and process-dependent, varying across restriction, reintroduction, and maintenance phases. Models that fail to encode the diet protocol, adherence assessment, and prediction horizon risk learning targets that are not transportable across settings or care pathways (23).

#### 3.4.2 Feature engineering: why taxonomy-only models rarely generalize

IBS responder models based solely on microbial relative abundance frequently show poor cross-cohort reproducibility. This reflects well-documented variability in IBS-associated microbiome patterns driven by geography, habitual diet, sequencing platforms, and confounding exposures such as medications or antibiotics (50, 51). Cross-cohort analyzes have explicitly demonstrated evidence of IBS-related microbiome signals, while also underscoring that these signatures vary substantially across populations, challenging the stability of taxa-based predictors (65).

For dietary response prediction, a multi-module feature architecture is therefore more plausible. Baseline microbiota ecology or subtype frameworks can provide contextual information relevant to diet responsiveness (48), but functional readouts—such as metabolomics, bile-acid profiles, and VOC or volatilome features—are often more directly linked to symptom-generating mechanisms and may generalize better than taxonomic features alone (43, 55, 56). Host susceptibility layers, including epithelial barrier integrity and neuroimmune activation markers, further modulate whether luminal exposures translate into symptoms and thus function as biologically meaningful moderators (44–47). Finally, high-resolution digital capture of dietary exposure and repeated symptom sampling is essential to reduce label noise and misclassification, particularly in free-living settings (Section 5.5) (59–63).

This organization aligns closely with methodological guidance from microbiome–ML reviews, which emphasize robust preprocessing, principled feature selection, and explicit confounder control over the pursuit of maximal dimensionality (66).

#### 3.4.3 Avoiding over-optimistic performance: preprocessing, confounding, and batch effects

Microbiome-based prediction models are especially vulnerable to experimental artifacts and analytic pitfalls. Foundational guidance has long emphasized the need for rigorous study design, standardized protocols, and artifact-aware pipelines to avoid false discoveries (67). In ML applications, batch effects can dominate model behavior, producing impressive internal discrimination that collapses when models are evaluated on external data.

Several methodological advances address this challenge directly. ConQuR (Conditional Quantile Regression for microbiome batch-effect removal) was developed to remove batch effects in microbiome data while accommodating zero inflation and compositional structure (68). Similarly, the SIAMCAT (Statistical Inference of Associations between Microbial Communities And host phenoTypes) toolbox was introduced to support cross-study comparative modeling with explicit safeguards against over-optimistic evaluation and unrecognized confounding (69). More recent benchmarking studies confirm that algorithm choice, preprocessing decisions, and batch-correction strategies have a substantial impact on external performance, and they advocate empirically validated pipelines over *ad hoc* analytical choices (70).

Collectively, these findings imply that IBS responder models should prespecify key analytic decisions, including batch-effect handling, confounder adjustment (e.g., medications, antibiotics, baseline diet patterns), leakage prevention through fully nested cross-validation, and harmonized metadata collection.

#### 3.4.4 Internal validation is not enough: external validation, transportability, and calibration

Even when discrimination appears acceptable internally, model performance commonly degrades in new settings. Targeted external validation—testing models in the intended population and care context rather than in convenience datasets—is therefore essential (71). A unifying framework for dataset shift and generalizability highlights how differences in case mix, measurement processes, and clinical workflows can destabilize prediction models and necessitate explicit documentation and, in many cases, model updating (72). Evidence across clinical prediction research consistently shows declines in both discrimination and calibration during external validation, underscoring the inadequacy of area under the receiver operating characteristic curve (AUC)-only reporting (73).

For IBS responder prediction, dataset shift is expected rather than exceptional. Habitual diet patterns, sequencing pipelines, symptom-reporting behavior, and dietary counseling workflows differ across regions and healthcare systems. A clinically credible modeling study should therefore incorporate geographic and, where possible, temporal external validation, alongside explicit calibration assessment and a predefined updating strategy—such as recalibration, transfer learning, or domain adaptation—when performance drift is detected (71, 72).

#### 3.4.5 Explainability that maps to biology: SHapley Additive exPlanations and module-level interpretation

For clinical adoption, model explanations must connect predictions to actionable biology rather than to long lists of unstable taxa. SHAP (SHapley Additive exPlanations) is widely used for *post hoc* interpretation of complex models, and recent guidance outlines best practices and limitations, particularly for binary outcomes and longitudinal settings relevant to diet–symptom trajectories (74).

In IBS precision nutrition, a two-level explainability strategy is especially useful. Local explanations address why a given individual is predicted to respond—for example, a combination of a high bile-acid module and diarrhea-predominant features suggesting benefit from a bile-acid–targeted strategy. Global explanations identify which biological modules most strongly influence response across populations, such as volatilome features reflecting fermentation regimes (43, 56). By operating at the module level, this approach preserves interpretability while remaining robust to cohort-specific taxonomic variation.

#### 3.4.6 Evaluating AI-guided diets as interventions: RCT evidence and reporting standards

A critical step beyond retrospective modeling is the evaluation of AI-guided dietary strategies as interventions. A recent multicenter randomized controlled trial compared a microbiome-based AI-assisted personalized diet with a low-FODMAP diet in IBS, demonstrating symptom improvement while emphasizing differences in microbiome diversity preservation between approaches (75). Such trials are pivotal because they test the entire AI pipeline—from sampling and modeling to dietary recommendation, adherence, and outcomes—rather than predictive accuracy in isolation.

To ensure transparency and reproducibility, AI intervention trials should follow established reporting standards. CONSORT-AI and SPIRIT-AI specify requirements for reporting human–AI interaction, input/output handling, and error analysis in trials involving AI systems (76, 77). For prediction model development and validation studies, TRIPOD+AI provides updated guidance, while PROBAST+AI strengthens risk-of-bias assessment across both regression-based and ML-based approaches (78, 79).

### 3.5 Clinical translation: embedding microbiome-informed prediction into IBS care pathways

#### 3.5.1 Where a prediction tool fits in real IBS workflows

In routine clinical practice, dietary management of IBS is typically escalated in a stepwise manner. General lifestyle and dietary advice is usually offered first, followed by more structured exclusion strategies—most notably low-FODMAP interventions—when symptoms persist and when appropriate dietary expertise is available (80, 81). This approach aligns closely with guideline recommendations, which consistently emphasize time-limited low-FODMAP trials delivered with supervision, structured reintroduction, and longer-term personalization, often under dietitian leadership (23, 33).

Within this context, a microbiome-informed prediction model should not be framed as a replacement for guideline-based care. Rather, its clinical role is best understood as a triage and optimization layer that supports decision-making at key points along the care pathway. Before initiating dietary intervention, prediction can be used to estimate the likelihood of response to different first-line options, including a structured low-FODMAP pathway, alternative dietary strategies such as low-carbohydrate or SSRD approaches, or an AI-personalized diet (28, 75). During intervention, early signals of non-response can prompt timely strategy adjustment or targeted adjunctive evaluation, such as assessment for bile-acid–driven diarrhea, methane-associated constipation, or the need for integrated gut–brain interventions. At the reintroduction and maintenance stages, prediction may support the definition of a personalized “minimal restriction set,” reducing long-term nutritional and ecological costs while preserving symptom control (23).

#### 3.5.2 The “minimum viable dataset” for clinically usable responder prediction

For clinical deployment, an IBS precision-nutrition model must function with a limited yet information-rich dataset, while allowing optional expansion to multi-omics when uncertainty remains high. Drawing on the mechanistic framework outlined in Sections 4–6, a minimum viable dataset should include four components.

First, core clinical phenotype data are indispensable, including Rome-based IBS subtype, baseline IBS-SSS, dominance of pain or bloating, stool frequency and form, symptom duration, and red-flag screening (1, 15). Second, exposure and behavioral context must be captured at sufficient resolution to reduce misclassification, encompassing baseline dietary pattern (in simplified form), major trigger foods, stress and sleep proxies, prior antibiotic or probiotic exposure, and adherence metrics during intervention (59–63).

Third, mechanism-proximal features with clear clinical actionability should be prioritized. Breath gas phenotyping (hydrogen and methane), when performed using standardized methodology, can reduce misclassification of fermentation- and transit-related phenotypes (20–22). In IBS-D–like presentations, assessment of bile-acid modules is particularly valuable because bile-acid diarrhea represents a treatable and mechanistically distinct subgroup (38, 40). Finally, an optional high-yield functional layer—such as targeted metabolite or VOC panels focusing on bile acids, tryptophan–indole pathways, or selected volatile compounds—can be added when available, as functional outputs often generalize better than taxonomic features across cohorts (43, 55, 56).

This staged dataset design supports progressive enhancement: low-burden inputs enable broad deployment, while additional layers are incorporated selectively when decision uncertainty justifies added complexity. To make the “minimum viable dataset” concept operational, we provide a stepwise worked example showing how core phenotype, exposure fidelity, and mechanism-proximal proxies can be encoded into model inputs, translated into interpretable module-level outputs, and linked to concrete clinical actions and switching rules. **Table 1** illustrates this end-to-end flow, emphasizing decision points aligned with routine IBS diet pathways.

**Table 1:** Worked example: feeding a minimum viable dataset into an IBS responder model (inputs, outputs, and clinical actions).

Step	What you collect (minimal dataset)	How it is encoded for the model	Model output (what it predicts)	What the clinician does next
1. Define baseline phenotype	Rome-based IBS subtype (IBS-D/IBS-C/IBS-M), symptom duration, red-flag screen; baseline IBS-SSS score (13)	IBS subtype as categorical; IBS-SSS as continuous; baseline stool frequency/form as continuous/ordinal	Baseline risk profile + “dominant symptom axis” (pain-dominant vs bowel-habit–dominant)	Confirms suitability for diet-first pathway; sets a prespecified endpoint target (IBS-SSS change threshold) and follow-up window (13–15)
2. Capture exposure with high fidelity	3-day diet record (or validated digital recall), trigger-food history, adherence plan (55)	Macro/micro summary + food-group features; FODMAP proxy features; adherence likelihood features	Predicted feasibility/adherence score and expected “restriction burden”	Chooses the least restrictive option among candidates when predicted efficacy is similar; documents planned intervention phases (21, 55)
3. Mechanism proxy: breath phenotype	Standardized hydrogen/methane breath test (when indicated) (18–20)	H_2_ and CH_4_ positivity/peak values; methane-linked phenotype flag	Probability that symptoms are fermentation-distension dominant vs methane-transit dominant	If methane phenotype is present, avoids treating the case as “simple IBS-C”; anticipates slower transit mechanisms and plans earlier switching if constipation persists (18–20, 34)
4. Functional panel (high-yield metabolites)	Targeted panel (examples): bile-acid synthesis/excretion markers when IBS-D-like (35, 38); optional targeted metabolite/VOC features when available (40, 51, 54)	Bile-acid module score; metabolite/VOC module scores; standardized z-scores	Mechanism module weights (e.g., bile-acid module high vs fermentation module high) and how much each module drives predicted response	If bile-acid module is high, prioritizes BA-aware diet structuring rather than prolonged broad restriction; treats the module as actionable stratification (35, 38)
5. Generate treatment probabilities	(No new data)	Model runs multi-class selection	Three response probabilities: low-FODMAP three-phase (21) vs low-carbohydrate/SSRD pathway (26, 29) vs microbiome-informed AI-personalized diet (if available) (10)	Picks the pathway with best balance of predicted response and sustainability; avoids over-restriction when alternatives are comparable (21, 26, 29)
6. Provide module-level explanation	(No new data)	Model explanation aggregated at module level (not taxa lists)	Top contributing modules (e.g., “fermentation module drives LFD response” or “bile-acid module predicts poor LFD response but better non-FODMAP strategy”)	Communicates a biologically interpretable rationale to support shared decision-making; documents why a pathway is chosen
7. Prespecify reassessment and switching	Scheduled reassessment at 4–6 weeks (restriction phase endpoint) (21)	Time horizon and switching rule encoded in protocol	Early non-response flag and switching recommendation consistent with adaptive/SMART logic (26, 29)	If no meaningful improvement at 4–6 weeks, switches rather than extending restriction; proceeds to alternate diet pathway or personalization (21, 26, 29)
8. Endpoint evaluation	IBS-SSS reassessment; pain + bowel habit composite aligned to regulatory guidance (13–15)	Outcome label for learning/quality improvement	Responder vs non-responder status (intervention-specific)	If responder: proceed to structured reintroduction and personalized maintenance (21); if non-responder: switch pathway and consider mechanism-directed workup (e.g., bile-acid evaluation, methane phenotype management) (34, 35, 38)

IBS, irritable bowel syndrome; IBS-SSS, Irritable Bowel Syndrome Severity Scoring System; H_2_, hydrogen; CH_4_, methane; VOC, volatile organic compounds; LFD, low-FODMAP diet; SSRD, starch- and sucrose-reduced diet; BA, bile acids; R&D, research and development; SMART, Sequential Multiple Assignment Randomized Trial.

#### 3.5.3 A pragmatic decision algorithm that aligns with guidelines

A clinically intuitive deployment strategy is a stratified decision algorithm that mirrors existing guideline pathways rather than competing with them. Initial triage should identify patients in whom severe pain, marked psychosocial comorbidity, or high distress signals dominate the clinical picture; for these individuals, integrated care models combining dietary and gut–brain interventions are likely to be more appropriate than repeated restrictive diets alone (1, 2, 33).

Mechanism-dominant flags can then guide targeted evaluation. Suspicion of bile-acid–driven diarrhea warrants assessment of bile-acid modules and consideration of bile-acid–focused strategies, including dietary fat patterning or sequestrants, given the distinct permeability and secretory mechanisms involved (38, 40). In constipation-predominant presentations, methane status should be treated as a modifier of transit physiology rather than relying solely on the IBS-C label, as methane-associated slowing represents a specific and actionable mechanism (37).

Diet selection follows from this stratification. When fermentation and luminal distension sensitivity dominate, a structured low-FODMAP pathway remains appropriate; when these features are less prominent, alternative dietary strategies such as low-carbohydrate or SSRD approaches may be preferable first-line options depending on phenotype and feasibility (28, 31). For individuals with high predicted benefit and a strong need for long-term sustainability, microbiome-based AI-personalized diets may be considered, supported by emerging randomized trial evidence (75). Importantly, this approach uses prediction to select the least restrictive effective option, rather than defaulting to maximal exclusion.

#### 3.5.4 Safety, sustainability, and the imperative to avoid over-restriction

While restrictive diets can improve IBS symptoms, prolonged or unnecessary exclusion carries risks. Broad fermentable carbohydrate restriction has been associated with reduced dietary diversity and depletion of beneficial taxa, including bifidobacteria, even in symptomatic responders (9). Accordingly, prediction tools should be designed explicitly to minimize over-restriction.

Key design principles include recommending time-limited trials with early response assessment, embedding structured reintroduction and personalization as the default endpoint rather than indefinite restriction (23), and favoring less restrictive dietary patterns when predicted efficacy is comparable, such as traditional dietary advice or Mediterranean-style patterns for selected patients (8, 27). From this perspective, the clinical value proposition of responder prediction is not solely higher response rates, but a reduction in unnecessary dietary burden and improved long-term adherence.

#### 3.5.5 Governance, privacy, and regulatory-grade development for AI-guided diets

Microbiome-informed precision nutrition tools occupy a space that increasingly resembles digital therapeutics or clinical decision support systems, bringing governance considerations to the forefront. Ethical frameworks articulated by the World Health Organization emphasize human rights, transparency, accountability, and inclusiveness as foundational principles for AI in health—issues that are particularly salient for microbiome-based personalization, where bias and inequitable access are realistic risks (82). More recent WHO guidance on large multi-modal models further reinforces governance requirements when generative or adaptive systems are used in clinical contexts (79).

Regulatory guidance on good machine learning practice (GMLP) highlights lifecycle management of AI/ML-based medical devices, including data representativeness, ongoing performance monitoring, and controlled change management—principles that translate directly to microbiome models, which are sensitive to shifts in diet culture and laboratory pipelines (80). In parallel, microbiome and dietary data constitute sensitive health information. Under GDPR and related frameworks, such data are classified as special category personal data, requiring enhanced safeguards and clearly defined lawful bases for processing (81, 82).

These considerations are not peripheral. They determine whether a responder prediction model can move beyond a single research environment and be deployed responsibly at scale. Taken together, successful clinical translation will depend on integrating mechanism-proximal measurements with governance-ready development and validation across settings.

### 3.6 Future directions: designing trials, validation gates, and mechanism-modular frameworks

#### 3.6.1 Designing the right trials: protocolized diets, adherence fidelity, and responder endpoints

The next step for IBS precision-nutrition research is unlikely to be a “better model” in the abstract. What will matter more is whether the intervention and outcomes are defined tightly enough that biological interpretation and cross-cohort prediction are even possible. In many studies, dietary exposure is not captured with sufficient fidelity: restriction is partial, reintroduction is unrecorded, and counseling intensity varies across participants. These forms of exposure noise blur mechanistic inference and make machine-learning models difficult to transport. A practical way forward is to treat the low-FODMAP diet as a staged intervention—restriction followed by structured reintroduction and personalized maintenance—with responder endpoints specified at prescheduled timepoints rather than collapsed into a single endpoint (23).

Responder definitions should remain clinically interpretable and anchored to validated instruments, such as meaningful changes in IBS-SSS, alongside regulator-aligned composite endpoints that emphasize improvement in abdominal pain together with bowel-habit outcomes (15, 18, 19). For AI-guided diets, the most informative evidence will come from pragmatic, multicenter randomized controlled trials that compare algorithm-guided personalization against guideline-adjacent dietary strategies delivered under real-world constraints. The recent multicenter trial comparing a microbiome-based AI-assisted personalized diet with a low-FODMAP diet is important in this respect because it evaluates the full clinical pathway—sampling, modeling, dietary recommendation, adherence, and outcomes—rather than treating prediction performance as the primary endpoint (75).

Several design choices can strengthen future trials. Stratified randomization by dominant mechanism signals (for example, bile-acid dysregulation or methane-associated slowing) can reduce heterogeneity dilution and clarify treatment effects (37, 38, 40). Adaptive and SMART-type designs are also well suited to IBS, where switching strategies after early non-response mirrors routine care and can be tested prospectively instead of being left to *post hoc* interpretation (28, 31, 75). In parallel, N-of-1 or crossover micro-trials, paired with high-resolution digital symptom tracking, can support trigger identification and individualized reintroduction while reducing recall bias (59–63).

#### 3.6.2 External validation as the gatekeeper: transportability, dataset shift, and calibration-first reporting

Clinical usefulness hinges on performance outside the development dataset. Targeted validation argues that external testing should occur in the intended population and care setting, rather than in convenient datasets with different case mix, workflows, or measurement processes (71). From a generalizability perspective, dataset shift is expected in IBS precision nutrition because habitual diet patterns, laboratory pipelines, symptom-reporting behavior, and delivery models differ across regions and health systems (72). Across clinical prediction research, both discrimination and calibration commonly worsen in external databases, making AUC-only reporting an incomplete and sometimes misleading summary of performance (73).

For microbiome-based features, these challenges are amplified by batch effects and analytic artifacts. Microbiome methodology guidance has shown how experimental and preprocessing choices can inflate false signals and erode reproducibility (67). ConQuR was developed to address batch effects under compositional and zero-inflated data structures common in microbiome studies (68), while SIAMCAT supports cross-study evaluation with explicit attention to confounding and generalization limits (69). Together, this implies that validation cannot be an afterthought: batch-effect handling, leakage prevention, and harmonized metadata need to be embedded from the start.

A pragmatic roadmap for IBS responder prediction should include at least one geographic external validation cohort and, where feasible, temporal validation. Reporting should prioritize calibration measures, including calibration-in-the-large and calibration slope, with recalibration considered when drift is observed. Benchmarking models with and without functional layers (for example, metabolite or VOC features) can test whether function-based signals improve portability beyond taxonomic profiles alone (55). Finally, intended use, required assays, and the target diet protocol should be documented explicitly so that a model’s output can be interpreted in clinical terms rather than as a decontextualized probability.

#### 3.6.3 Moving from taxa to actionable mechanism modules

A durable precision framework for IBS should be organized around mechanism modules that map to clinical decisions and can be measured in multiple ways. This is partly pragmatic: microbial taxa differ substantially across cohorts, whereas functional outputs and host susceptibility traits may converge more reliably.

A fermentation and luminal distension module is supported by controlled physiology studies showing that poorly absorbed short-chain carbohydrates increase delivery of water and fermentable substrates to the colon and alter gas production patterns linked to symptom generation (35, 36). Volatilomic profiling provides a functional readout of this metabolic regime, and fecal volatome signatures connected to IBS metabotypes have been associated with response to low-FODMAP intervention, supporting their use as interpretable intermediate phenotypes (43).

A bile-acid module captures a clinically actionable subgroup within IBS-D characterized by increased bile-acid synthesis or excretion and distinct physiological features (38). The association between bile-acid diarrhea and increased intestinal permeability further suggests that epithelial stress may be integral to this module rather than an unrelated abnormality (40). Barrier-related mechanisms form a separate but overlapping module: increased permeability has been linked to hypersensitivity in IBS-D (44), and more granular epithelial studies indicate subtype-specific features, including tricellular tight junction dysfunction in IBS-M (46).

Neuroimmune amplification represents an additional layer. Activated mast cells in proximity to enteric nerves correlate with abdominal pain, implicating immune–neural interactions in symptom generation (47). Complementary evidence indicates that the microbiota can modulate serotonin handling through a mast cell–PGE_2_ pathway, providing a plausible bridge between luminal ecology, motility, and pain signaling (41). Translationally, the priority is not to expand biomarker lists indefinitely, but to define scalable, targeted readouts for each module that can be implemented across sites and used for explainable modeling.

To translate the modular framework into practice, the key step is to align each mechanism module with trial-ready dietary pathways and outcome anchors that are reproducible across sites. We therefore summarize landmark human IBS dietary studies spanning tightly controlled efficacy trials to pragmatic implementation and digital delivery, highlighting design features, endpoints, and adherence considerations that materially affect transportability. **Table 2** provides these trial anchors and deployable pathways to support both study design and external validation.

**Table 2:** Trial anchors and deployable dietary pathways for IBS (from controlled efficacy to deployable personalization).

Intervention type	Study (author, year, journal)	Design/population	IBS subtype(s)	Intervention vs comparator	Duration	Primary endpoint(s) (examples)	Key take-home message	Implementation/adherence notes	Ref.
Low-FODMAP (controlled feeding)	Halmos, 2014, *Gastroenterology*	Randomized crossover; controlled feeding	Mixed IBS	Low-FODMAP vs habitual/typical diet	~3 weeks per phase	Global GI symptom score; symptom severity	High internal validity “anchor” showing symptom reduction under tightly controlled exposure	Best for mechanistic anchoring (minimizes exposure noise); less representative of routine care	(7)
Low-FODMAP vs traditional advice	Böhn, 2015, *Gastroenterology*	RCT; outpatient dietitian-led	Mixed IBS	Low-FODMAP vs traditional IBS dietary advice	~4 weeks	IBS symptom outcomes (e.g., IBS-SSS)	Both pathways can improve symptoms; supports “multiple first-line pathways + responder selection” rather than single-diet dominance	Counseling intensity and adherence monitoring materially affect outcomes	(8)
Low-FODMAP (IBS-D, QoL outcomes)	Eswaran, 2016, *Am J Gastroenterol*	RCT; outpatient	IBS-D	Low-FODMAP vs conventional dietary advice	~4 weeks	QoL; activity impairment; symptom outcomes	Demonstrates clinically meaningful patient-centered benefit beyond symptom scores	Useful “value” anchor for real-world uptake; track adherence and reintroduction planning	(24)
Low-FODMAP: ecology trade-off	Staudacher, 2012, *J Nutr*	Dietary restriction study	IBS	Fermentable carbohydrate restriction	—	Microbiota shifts + symptoms	Symptom improvement may accompany reductions in beneficial taxa (e.g., bifidobacteria), motivating precision selection and staged reintroduction	Reinforces the need for phase-based LFD delivery and monitoring	(9)
Mediterranean diet vs Low-FODMAP	Singh, 2025, *Neurogastroenterol Motil*	RCT	Non-constipated IBS	Mediterranean diet vs low-FODMAP	—	Symptom/QoL outcomes	Shows that less restrictive “diet quality” pathways can be competitive in some populations	Supports offering least-restrictive effective option first, then switching if needed	(27)
Low-FODMAP vs low-carb vs pharmacologic care	Nybacka, 2024, *Lancet Gastroenterol Hepatol*	RCT; comparative strategies	IBS	Low-FODMAP vs low-carbohydrate diet vs drug therapy	—	Symptom outcomes	Head-to-head comparative evidence supports a portfolio approach (choose among pathways)	Natural evidence base for “switching rules” and adaptive strategies	(28)
Starch- & sucrose-reduced diet (SSRD)	Nilholm, 2021, *Nutrients*	RCT	Mixed IBS	SSRD vs habitual diet control	4 weeks	IBS-SSS (responder threshold)	Non-FODMAP pathway with clear symptom benefit; useful alternative when LFD is poorly tolerated	Strong for implementation: simple rules; monitor carb quality and sustainability	(31)
SSRD (systemic mediators)	Roth, 2022, *Nutrients*	RCT/mechanistic outcomes	IBS	SSRD vs control	—	Symptoms + metabolic/inflammatory mediators	Adds a “system readout” layer that can map onto mechanism modules (metabolic/inflammatory traits)	Good bridge into metabolomics-informed stratification	(32)
Gluten-free diet (IBS-D physiology)	Vazquez-Roque, 2013, *Gastroenterology*	Controlled trial	IBS-D	Gluten-free vs gluten-containing	~4 weeks	Stool frequency; intestinal function	Suggests a responsive subgroup; highlights confounding by wheat components (e.g., fructans)	Use as “selective pathway” rather than blanket recommendation	(29)
Metabolic profiling for LFD responsiveness	Algera, 2022, *Aliment Pharmacol Ther*	Prediction-focused clinical study	IBS	Metabolomics as predictor of LFD response	—	Response prediction	Functional features (metabolome) can outperform taxonomy-only signals for response stratification	Supports “function-first” modeling layer in your framework	(30)
Microbiome subtype stratification	Vervier, 2022, *Gut*	Subtyping + diet response	IBS	Baseline microbiota subtype vs LFD response	—	Symptom response by subtype	Microbiota states act as effect modifiers → conceptual basis for subtype-aware prediction models	Use to justify feature design + external validation need	(10)
AI-assisted personalized diet vs Low-FODMAP	Tunali, 2024, *Am J Gastroenterol*	Multicenter RCT	Mixed IBS	Microbiome/AI-guided diet vs low-FODMAP	6 weeks	IBS-SSS; symptom relief	“End-to-end” pipeline can be evaluated pragmatically against guideline-adjacent care	Emphasize sampling logistics, adherence, and interpretability as trial-critical	(12)
Digital/app-based dietary care	Rafferty, 2021, *JMIR mHealth uHealth*	RCT (digital intervention)	IBS	App-based diet program vs comparator	—	Symptoms/QoL outcomes	Digital delivery enables higher-frequency symptom capture and scalable implementation	Best positioned as adherence + phenotyping infrastructure for SMART/N-of-1 designs	(60)
Digital health evidence base	D’Silva, 2024, *Dig Dis Sci*	Systematic review	IBS	Digital interventions	—	Symptoms/self-management outcomes	Supports feasibility/benefit of digital support as a delivery layer	Use as “infrastructure” evidence, not as mechanistic proof	(59)

IBS, irritable bowel syndrome; FODMAP, fermentable oligosaccharides, disaccharides, monosaccharides and polyols; LFD, low-FODMAP diet; RCT, randomized controlled trial; QoL, quality of life; IBS-SSS, Irritable Bowel Syndrome Severity Scoring System; SSRD, starch- and sucrose-reduced diet; AI, artificial intelligence; JMIR, Journal of Medical Internet Research.

Building on the trial anchors above, we operationalize the framework as a set of mechanism modules mapped to minimal, clinically actionable proxies, first-line diet pathways, and prespecified switching rules. This mapping is intentionally “minimum deployable,” prioritizing mechanism-proximal readouts and time-limited restriction with early reevaluation. **Table 3** summarizes the module-to-pathway map, switching triggers, and endpoints suitable for both routine care and validation-first trials.

**Table 3:** From mechanism modules to actionable diet pathways in IBS: minimal proxies, switching rules, and endpoints.

Module	Minimal proxies (what to measure first)	First-line diet pathway (start here)	When to switch (rule-based)	Primary endpoint (how to judge response)
Fermentation/luminal distension	Postprandial bloating/gas with carbohydrate-trigger history; when indicated, standardized hydrogen/methane breath testing to improve phenotyping (20–22); mechanistic support that poorly absorbed short-chain carbohydrates increase small-intestinal water/substrate delivery and alter gas-production patterns (25, 26); when feasible, functional readouts such as fecal volatilomics/volatilome for a more proximal metabolic signal (42, 43)	Low-FODMAP three-phase pathway (restriction, structured reintroduction, personalized maintenance) as the default structured approach (23); in resource-limited settings, structured “traditional IBS dietary advice” can serve as an alternative first-line pathway (8); for non-constipated IBS, a Mediterranean-style pattern may offer a more sustainable, diversity-preserving option (27)	If there is no clinically meaningful improvement after 4–6 weeks of the restriction phase (or improvement fails to meet prespecified thresholds), switch rather than prolong broad restriction (23); consider switching to low-carbohydrate or SSRD-type strategies (28, 31), or to AI-personalized diet pathways when available (12)	Clinically meaningful change in IBS-SSS (15), aligned with regulator-oriented composite endpoints emphasizing abdominal pain improvement together with bowel habit improvement (18, 19)
Methane–transit slowing	Standardized breath testing demonstrating methane positivity (20–22); constipation/straining and stool form-frequency patterns consistent with slowed transit; experimental evidence that methane can slow intestinal transit and modulate contractile activity (37)	Do not treat this as “IBS-C label” alone; manage as a methane-linked transit phenotype. Use a diet pathway that limits unnecessary broad restriction while targeting gas-related triggers and moving promptly to individualized maintenance (23, 37); comparator pathways include low-carbohydrate or SSRD-type strategies (28, 31)	If bloating improves but constipation/defecatory difficulty does not improve by 4–6 weeks, consider the transit module as unresolved and switch to an alternative diet pathway and/or add mechanism-directed adjunct management rather than escalating restriction (23, 28, 31, 37)	IBS-SSS (15) plus abdominal pain and bowel habit composite improvement (18, 19), avoiding reliance on bloating alone when constipation remains dominant
Bile-acid module	In IBS-D-like presentations with urgency and watery stools, consider bile-acid–related mechanisms; markers of increased bile acid synthesis/excretion support subgrouping (38, 39); bile acid diarrhea is associated with increased permeability, suggesting barrier stress can be coupled to this module (40); metabolomics layers may further support bile-acid–linked functional readouts (56)	Avoid defaulting to fermentation-only pathways. Prioritize bile-acid–oriented dietary structuring (e.g., individualized fat patterning and timing) and avoid prolonged broad restriction; if fermentation triggers are also prominent, a low-FODMAP three-phase approach can be used with earlier reintroduction emphasis (23, 38, 40)	If pain improves modestly but diarrhea/urgency does not improve by 4–6 weeks, reclassify the dominant mechanism and switch toward bile-acid–targeted pathways or alternative diets rather than continuing broad restriction (23, 38, 40); comparator pathways include low-carbohydrate or SSRD-type strategies (28, 31)	IBS-SSS (15) and regulator-aligned composite endpoints (18, 19), with parallel tracking of stool frequency/urgency and Bristol stool form as key mechanistic outcomes
Barrier susceptibility	Treat as a host susceptibility layer: increased permeability associates with hypersensitivity (44), with subtype-specific epithelial alterations reported (e.g., tricellular tight junction dysfunction in IBS-M) (46); clinically, pain-dominant presentations with low symptom thresholds can function as pragmatic susceptibility cues (not diagnostic tests) (44–46)	Prefer sustainability and avoid over-restriction. If low-FODMAP is used, adhere to the three-phase protocol with timely reintroduction to limit ecological/nutritional costs (23); for non-constipated IBS, Mediterranean-style patterns may be appropriate for diversity preservation (27)	If global symptoms remain largely unchanged at 4–6 weeks, especially with persistent pain dominance, switch to alternative diet pathways or broader multimodal management rather than intensifying restriction (23, 28, 31)	IBS-SSS (15) and composite endpoints (18, 19); document susceptibility features as effect modifiers to support interpretation rather than attributing failure to adherence alone
Neuroimmune amplification	Mast-cell proximity to nerves correlates with abdominal pain (47); microbiota can modulate serotonin handling via a mast cell–PGE_2_ pathway linking luminal ecology to motility and pain signaling (41); in research settings, functional metabolite layers can support interpretation of amplification phenotypes (41, 56)	Use diet pathways that reduce triggers while preserving sustainability: low-FODMAP three-phase with early reintroduction (23), or SSRD/low-carbohydrate comparators when appropriate (28, 31); when available, microbiome-informed AI-personalized diets can be considered in complex, multi-trigger cases (12)	If abdominal pain does not reach clinically meaningful improvement by 4–6 weeks, switch diet strategy and consider integrating a broader DGBI-focused care package rather than repeated escalation of restriction (23, 28, 31)	IBS-SSS (15) and regulator-aligned composite endpoints (18, 19), with explicit reporting of pain dimensions to reflect the amplification module

IBS, irritable bowel syndrome; IBS-D, diarrhea-predominant IBS; IBS-C, constipation-predominant IBS; IBS-M, mixed bowel habits IBS; H_2_, hydrogen; CH_4_, methane; VOCs, volatile organic compounds; LFD, low-FODMAP diet; SSRD, starch- and sucrose-reduced diet; BA, bile acids; DGBI, disorders of gut–brain interaction; FDA, U.S. Food and Drug Administration; EMA, European Medicines Agency; PGE_2_, prostaglandin E_2_; SERT, serotonin reuptake transporter.

#### 3.6.4 Reporting standards, risk of bias, and regulatory-grade reproducibility

To avoid a proliferation of models that look strong internally but fail in practice, reporting and governance standards should be treated as part of scientific validity. TRIPOD+AI provides updated guidance for reporting prediction model development and validation using regression or machine-learning methods (78), while PROBAST+AI strengthens assessment of risk of bias and applicability with explicit attention to AI-specific failure modes (79). For randomized trials evaluating AI-based dietary interventions, CONSORT-AI and SPIRIT-AI specify requirements relevant to human–AI interaction, data handling, and error analysis (76, 77).

Beyond reporting, regulatory guidance increasingly emphasizes lifecycle management. Good machine learning practice principles highlight representative data, continuous performance monitoring, and controlled updating—issues that are especially relevant for microbiome-based tools vulnerable to assay drift and shifts in dietary culture (80). Methodologically, preregistration of modeling plans, release of code and pipelines, publication of de-identified feature tables where permitted, and routine reporting of calibration and decision-curve utility alongside discrimination should be treated as baseline expectations rather than optional enhancements.

## 4 Discussion and summary

**Figure 1:**
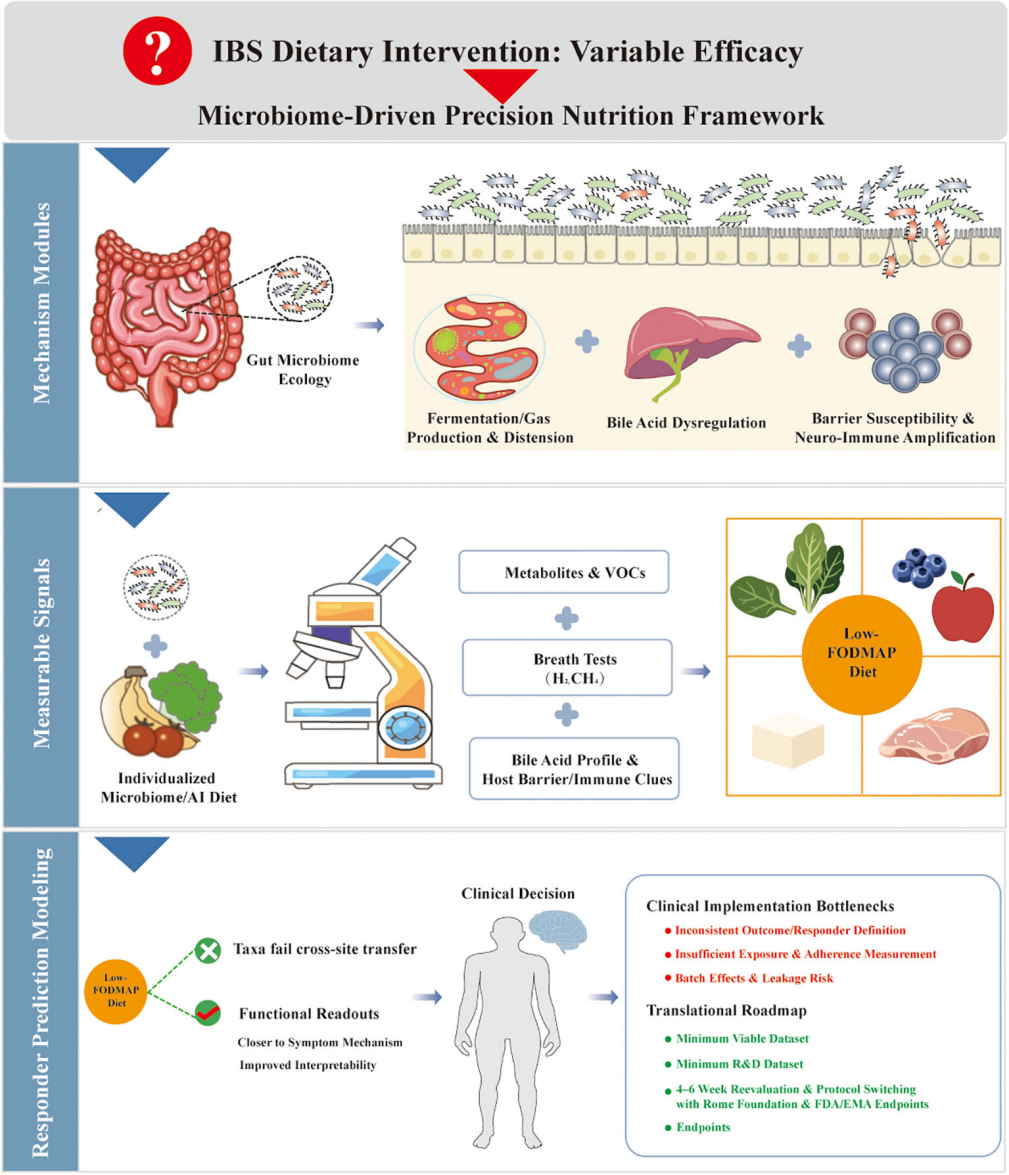
Microbiome-driven precision nutrition framework for IBS dietary intervention.

Irritable bowel syndrome (IBS) is a prototypical disorder of gut–brain interaction characterized by marked heterogeneity in symptom drivers and treatment response (1–4). Dietary therapy is widely used and often effective, yet responses are inconsistent and intervention delivery is frequently non-uniform, limiting mechanistic interpretation and cross-cohort generalizability (7, 8, 23). In this conceptual analysis, we argue that “microbiome-informed precision nutrition” becomes unavoidable in IBS not because a single diet is universally superior, but because baseline host–microbe–diet configurations appear to modify treatment effects and can be measured in ways that are increasingly actionable. **Figure 1** summarizes the proposed microbiome-driven precision nutrition framework, linking mechanism modules, measurable signals, responder prediction, and clinical implementation constraints. Evidence supporting microbiome subtyping and microbiome-assisted personalization illustrates that diet response is, at least in part, stratifiable rather than purely stochastic (10–12).

We synthesize mechanistic and translational evidence into a feature-ready framework that prioritizes mechanism-proximal signals over unstable taxonomic lists. Specifically, diet-related symptom change can be interpreted through modular pathways that include fermentation/distension physiology (25, 26, 35, 36), bile-acid dysregulation as a treatable IBS-D mechanism (38, 40), and host susceptibility layers such as epithelial barrier dysfunction and neuroimmune amplification (44–47), with functional readouts (metabolites and volatilomics) offering potential advantages for portability across cohorts (43, 55, 56). We further outline how these modules can be operationalized into prediction targets, feature stacks, and validation plans that fit real-world IBS workflows, emphasizing that clinical utility depends on protocolized dietary exposure, high-fidelity adherence measurement, and targeted external validation with calibration-first reporting (15, 18, 19, 59–63, 71–73). Finally, we highlight that reporting standards and lifecycle governance are not ancillary; they are prerequisites for reproducible and deployable responder models and for RCTs evaluating AI-guided diet interventions (76–79, 83).

## 5 Conclusion

IBS precision nutrition is best viewed as a responder-prediction problem embedded in a staged clinical diet pathway, rather than as a search for a universally optimal diet. The field’s central task is to align dietary interventions with measurable mechanism modules and to predict which patients are likely to benefit, which require adjunctive evaluation (for example, bile-acid or methane-linked phenotyping), and which should avoid unnecessary restriction and proceed directly to alternative strategies (23, 37, 38, 40). Progress will depend less on incremental model complexity and more on disciplined definitions: intervention-specific responder endpoints, standardized exposure and adherence capture, and explicit time horizons that reflect restriction, reintroduction, and maintenance phases (15, 18, 19, 23, 59–63).

For modeling, the most durable path is modular: integrate baseline microbial ecology with functional outputs and host susceptibility signals, then evaluate transportability through targeted external validation and calibration, not internal AUC alone (43, 55, 67–73). For translation, the practical benchmark is end-to-end evidence: pragmatic multicenter trials that test the full pipeline from measurement to recommendation to outcome, reported under modern AI-specific standards and governed through lifecycle monitoring and controlled updating (12, 75–79, 83). Under these conditions, microbiome-informed precision nutrition can shift IBS dietary care from trial-and-error toward mechanism-guided, minimally restrictive personalization that is clinically interpretable, reproducible across settings, and ultimately scalable.
