# Supplementary material for: Reframing precision nutrition in irritable bowel syndrome: a mechanism-informed conceptual framework for responder prediction and clinical translation
Source: Front Immunol. 2026 May 29;17:1809221. doi: 10.3389/fimmu.2026.1809221 (PMC13259811; doi:10.3389/fimmu.2026.1809221)
Supplement: Supplementary file 1 [file Table1.docx]

**Supplementary Table S1. Key Studies and Evidence Sources Informing the Precision Nutrition Framework**

Studies are organized by manuscript section. Reference numbers in square brackets correspond to the main text citation list. The 'Identification Source' column reports whether each study was retrieved via PubMed/MEDLINE database searching, citation tracking from key consensus statements or pivotal trials, or hand-searching of regulatory and guideline databases. PubMed/MEDLINE searches were conducted from database inception through March 2025; citation tracking extended through February 2026. This table is not exhaustive: it catalogues the pivotal, methodologically distinctive, or operationally defining studies cited as primary evidence for each section. A complete reference list is provided in the main manuscript.

Abbreviations: IBS, irritable bowel syndrome; IBS-D/C/M, IBS with predominant diarrhea/constipation/mixed bowel habits; RCT, randomized controlled trial; ML, machine learning; FODMAP, fermentable oligosaccharides, disaccharides, monosaccharides and polyols; SSRD, starch- and sucrose-reduced diet; VOC, volatile organic compound; BA, bile acid; SERT, serotonin reuptake transporter; GI, gastrointestinal; DGBI, disorder of gut–brain interaction; GDPR, General Data Protection Regulation; SHAP, SHapley Additive exPlanations; ConQuR, Conditional Quantile Regression; SIAMCAT, Statistical Inference of Associations between Microbial Communities And host phenoTypes.

| **Section** | **Study Design** | **First Author [Ref]** | **Year** | **Journal / Source** | **Identification Source** | **Key Finding / Relevance to Framework** |
| --- | --- | --- | --- | --- | --- | --- |
| Sec 2 | Clinical guideline | Drossman DA [1,2] | 2016/17 | Gastroenterology; J Neurogastroenterol Motil | Citation tracking | Rome IV diagnostic criteria and IBS subtype definitions (IBS-D, IBS-C, IBS-M) |
| Sec 2 | Epidemiological study | Sperber AD [4] | 2021 | Gastroenterology | PubMed/MEDLINE | Worldwide DGBI prevalence >40%; quality-of-life and healthcare burden |
| Sec 2 | Guideline | Lacy BE [31] | 2021 | Am J Gastroenterol | Citation tracking | ACG clinical guideline: low-FODMAP recommendation; dietitian-led implementation |
| Sec 2 | Regulatory document | U.S. FDA [14] | 2012 | FDA Guidance | Hand-searched (regulatory/guideline) | Endpoint framework: abdominal pain and bowel habit as core IBS trial domains |
| Sec 2 | Regulatory document | EMA [15] | 2014 | EMA Guideline CPMP/EWP/785/97 | Hand-searched (regulatory/guideline) | European endpoint recommendations for IBS treatment evaluation |
| Sec 2 | Validated instrument | Francis CY [13] | 1997 | Aliment Pharmacol Ther | PubMed/MEDLINE | IBS Severity Scoring System (IBS-SSS): validation and minimally important difference |
| Sec 2 | Consensus | Gasbarrini A [18] | 2009 | Aliment Pharmacol Ther | Citation tracking | Rome Consensus: indications and methodology for H2 breath testing in GI disorders |
| Sec 2 | Consensus | Rezaie A [19] | 2017 | Am J Gastroenterol | Citation tracking | North American Consensus: H2/CH4 breath testing standardization (substrate, dosing, interpretation) |
| Sec 2 | Guideline | Hammer HF [20] | 2022 | United Eur Gastroenterol J | PubMed/MEDLINE | European guideline: comprehensive breath-testing framework for adults and pediatric populations |
| Sec 2 | Reporting standard | Page MJ (PRISMA 2020) [11] | 2021 | BMJ | Citation tracking | PRISMA 2020: updated guideline for reporting systematic reviews |
| Sec 2 | Reporting standard | Tricco AC (PRISMA-ScR) [12] | 2018 | Ann Intern Med | Citation tracking | PRISMA extension for scoping reviews (PRISMA-ScR): checklist and explanation |
| Sec 3 | RCT | Halmos EP [5] | 2014 | Gastroenterology | PubMed/MEDLINE | Low-FODMAP vs habitual diet; significant reduction in global IBS symptoms |
| Sec 3 | RCT | Böhn L [6] | 2015 | Gastroenterology | PubMed/MEDLINE | Low-FODMAP vs traditional dietary advice; comparable symptom reduction in both arms |
| Sec 3 | RCT | Eswaran S [22] | 2016 | Am J Gastroenterol | PubMed/MEDLINE | Low-FODMAP vs mNICE: 52% vs 21% adequate relief (p=0.0001); superior abdominal pain response |
| Sec 3 | Controlled study | Staudacher HM [7] | 2012 | J Nutr | PubMed/MEDLINE | FODMAP restriction reduces luminal bifidobacteria; symptom-ecology trade-off |
| Sec 3 | Review/practice guidance | Lomer MCE [21] | 2024 | Proc Nutr Soc | PubMed/MEDLINE | Long-term low-FODMAP management; structured reintroduction; dietitian-led delivery |
| Sec 3 | RCT | Singh P [25] | 2025 | Neurogastroenterol Motil | PubMed/MEDLINE | Mediterranean diet vs low-FODMAP: symptom improvement in both; differences in feasibility and long-term acceptability |
| Sec 3 | Multi-arm RCT | Nybacka S [26] | 2024 | Lancet Gastroenterol Hepatol | PubMed/MEDLINE | Low-FODMAP vs low-carbohydrate vs pharmacotherapy; differential benefit across patients |
| Sec 3 | Controlled trial | Vazquez-Roque MI [27] | 2013 | Gastroenterology | PubMed/MEDLINE | Gluten-free diet in IBS-D; bowel frequency and intestinal function |
| Sec 3 | RCT | Nordin E [29] | 2021 | Nutrients | PubMed/MEDLINE | SSRD (starch- and sucrose-reduced diet) vs comparison: 4-week symptom improvement with metabolic changes |
| Sec 4 | Controlled feeding study | Barrett JS [23] | 2010 | Aliment Pharmacol Ther | PubMed/MEDLINE | Poorly absorbed short-chain carbohydrates increase water and fermentable substrate delivery to proximal colon |
| Sec 4 | Controlled feeding study | Ong DK [24] | 2010 | J Gastroenterol Hepatol | PubMed/MEDLINE | Dietary FODMAP manipulation alters H2/CH4 breath profiles and reproduces GI symptoms in IBS |
| Sec 4 | Mechanistic study | Pimentel M [32] | 2006 | Am J Physiol Gastrointest Liver Physiol | PubMed/MEDLINE | Methane produced by enteric bacteria slows intestinal transit; mechanistic basis for IBS-C phenotype |
| Sec 4 | Mechanistic cohort | Camilleri M [33] | 2014 | Am J Gastroenterol | PubMed/MEDLINE | Increased bile-acid synthesis or fecal excretion defines clinically actionable IBS-D subgroup |
| Sec 4 | Cohort study | Wong BS [34] | 2012 | Clin Gastroenterol Hepatol | PubMed/MEDLINE | Increased bile-acid biosynthesis associated with IBS-D; supports BA as effect modifier |
| Sec 4 | Mechanistic study | Gao J [35] | 2022 | Gastroenterology | PubMed/MEDLINE | Gut microbiota modulates SERT via mast cell–PGE2 pathway; links microbial activity to IBS neurochemical regulation |
| Sec 4 | Cohort study | Magnus Y [36] | 2022 | Gastroenterology | PubMed/MEDLINE | Bile-acid diarrhea associated with increased intestinal permeability vs IBS-D without bile-acid malabsorption |
| Sec 4 | Observational study | Ahmed I [37] | 2013 | PLoS One | PubMed/MEDLINE | Fecal VOC profiles discriminate IBS from IBD and healthy controls |
| Sec 4 | Cohort + intervention | Conley TE [38] | 2024 | EBioMedicine | PubMed/MEDLINE | Volatilome-defined metabotypes predict low-FODMAP response; VOC shifts track symptom improvement in responders |
| Sec 4 | Observational cohort | Zhou QQ [39] | 2009 | Pain | PubMed/MEDLINE | Increased intestinal permeability associated with hypersensitivity and symptom severity in IBS |
| Sec 4 | Observational study | Ludidi S [40] | 2015 | PLoS One | PubMed/MEDLINE | Intestinal barrier dysfunction in IBS: multiple contributing pathways, particularly in IBS-D |
| Sec 4 | Mechanistic cohort | Awad K [41] | 2023 | Cells | PubMed/MEDLINE | Impaired tricellular tight-junction function and altered antigen uptake in IBS-M (ex vivo) |
| Sec 4 | Observational study | Barbara G [42] | 2004 | Gastroenterology | Citation tracking | Activated mast cells near colonic nerves correlate with abdominal pain severity in IBS |
| Sec 5 | Systematic review | Pittayanon R [43] | 2019 | Gastroenterology | PubMed/MEDLINE | Gut microbiota in IBS: systematic review; limited cross-study reproducibility of taxonomic signatures |
| Sec 5 | Population cohort | Hugerth LW [44] | 2020 | Gut | PubMed/MEDLINE | No distinct microbiome signature for IBS in Swedish random population; cautions against taxa as stable markers |
| Sec 5 | Cohort study | Tap J [45] | 2017 | Gastroenterology | PubMed/MEDLINE | Intestinal microbiota signature associated with IBS severity; microbial community state as physiologically relevant |
| Sec 5 | Cohort + dietary intervention | Vervier K [8] | 2022 | Gut | PubMed/MEDLINE | Two microbiota subtypes in IBS with distinct low-FODMAP responses; baseline community structure as effect modifier [KEY stratification study] |
| Sec 5 | Prospective intervention | Wilson B [48] | 2023 | Aliment Pharmacol Ther | PubMed/MEDLINE | Fecal and urine metabolites—but not gut microbiota composition—predict low-FODMAP response in IBS |
| Sec 5 | Review | Bennet SM [49] | 2020 | Neurogastroenterol Motil | PubMed/MEDLINE | Metabolomics in IBS: altered bile acid, SCFA, and amino acid pathways; analytical standardization needs |
| Sec 5 | Crossover RCT | Nordin E [51] | 2023 | Am J Physiol Regul Integr Comp Physiol | PubMed/MEDLINE | FODMAP challenge alters bile acids, phenolic- and tryptophan-derived metabolites; mechanistic link to motility and gut–brain signaling |
| Sec 5 | ML cohort study | Fukui H [46] | 2020 | J Clin Med | PubMed/MEDLINE | ML-based gut microbiome analysis for IBS identification; multivariate patterns vs univariate taxa |
| Sec 5 | ML study | Su Q [47] | 2022 | Nat Commun | PubMed/MEDLINE | Faecal microbiome-based ML for multi-class disease diagnosis; cross-cohort performance considerations |
| Sec 6 | Cross-cohort ML study | Li J [58] | 2024 | Adv Sci | PubMed/MEDLINE | Cross-cohort microbiome signatures for IBS presentation and treatment; transportability of ML signals |
| Sec 6 | Methods paper | Kim D [60] | 2017 | Microbiome | PubMed/MEDLINE | Pitfalls in microbiome research: need for standardized protocols and artifact-aware analytical pipelines |
| Sec 6 | Methods paper (ConQuR) | Ling W [61] | 2022 | Nat Commun | PubMed/MEDLINE | Conditional Quantile Regression (ConQuR): batch-effect removal for microbiome data accommodating zero inflation and compositional structure |
| Sec 6 | Methods paper (SIAMCAT) | Wirbel J [62] | 2021 | Genome Biol | PubMed/MEDLINE | SIAMCAT toolbox for cross-study microbiome ML with safeguards against over-optimistic evaluation and unrecognized confounding |
| Sec 6 | Methods paper | Li P [63] | 2025 | Gut Microbes | PubMed/MEDLINE | Best practices for microbiome-based ML disease classifiers; analytic decision prespecification |
| Sec 6 | Validation framework | Sperrin M [64] | 2022 | Diagn Progn Res | PubMed/MEDLINE | Targeted external validation in intended population and setting; calibration alongside discrimination |
| Sec 6 | Methods paper (SHAP) | Ponce-Bobadilla AV [67] | 2024 | Clin Transl Sci | PubMed/MEDLINE | SHAP (SHapley Additive exPlanations): best practices for explainability of supervised ML models |
| Sec 6 | Reporting standard | Collins GS (TRIPOD+AI) [68] | 2024 | BMJ | Citation tracking | Updated reporting guidelines for clinical prediction models using regression or ML methods |
| Sec 7–8 | Pilot RCT | Karakan T [9] | 2022 | Gut Microbes | PubMed/MEDLINE | AI-driven personalized diet in IBS-M: reduced symptom severity with shifts in selected taxa |
| Sec 7–8 | Multicenter RCT | Tunali V [10] | 2024 | Am J Gastroenterol | PubMed/MEDLINE | Microbiome-based AI-personalized diet vs low-FODMAP: multicenter head-to-head RCT; higher-level evidence for algorithm-guided personalization |
| Sec 7–8 | Reporting standard | Liu X (CONSORT-AI) [70] | 2020 | Nat Med | Citation tracking | CONSORT-AI extension for reporting clinical trials of AI interventions |
| Sec 7–8 | Reporting standard | Cruz Rivera S (SPIRIT-AI) [71] | 2020 | Nat Med | Citation tracking | SPIRIT-AI extension for clinical trial protocols involving AI |
| Sec 7–8 | WHO guidance | WHO [74,75] | 2021/25 | WHO Publications | Hand-searched (regulatory/guideline) | Ethics and governance of AI for health; large multi-modal models; data protection and equity considerations |
| Sec 7–8 | Regulatory document | EU GDPR [77] | 2016 | Official J EU | Hand-searched (regulatory/guideline) | GDPR Article 9: processing of special categories of personal data; relevant to microbiome data governance |
